# High-quality contact tracing, case investigation, and isolation of suspected and confirmed cases: a key strategy to control the spread of infection

**DOI:** 10.2478/abm-2023-0049

**Published:** 2023-10-09

**Authors:** Editorial Office of Asian Biomedicine

**Affiliations:** 1 Faculty of Medicine, Chulalongkorn University, Bangkok 10330, Thailand

In order to control the spread of infections, contact tracing is a key strategy for early tracing of contacts, case investigation of suspected or confirmed infection as well as isolation of infected cases [1]. It is important that epidemiologists or public health workers work with infected cases to recall everyone they have close contact within the period that the cases might be able to transmit the infections. The results of a systematic review and meta-analysis suggest that a physical distancing of at least 1 m may be used as a criterion to estimate whether the contacts with the suspected or confirmed cases warrant investigations for infections [2]. Confidentiality must be strictly adhered to, to protect the privacy and confidentiality of infected cases and contacts, particularly when multiple counseling sessions are required [3]. If possible, the identity of infected cases to whom the contacts may have been exposed is to be kept confidential.

Contacts should be informed about the need to isolate themselves from those who are not exposed. They should also be advised to continuously monitor their symptoms. They should be reminded that they may spread the infections if they harbor the virus even though they are asymptomatic. They are encouraged to stay separate from those who are not exposed, the so-called social distancing, at least 6 feet for a period of about 2 weeks after their last exposure to infected individuals. Some innovative methods to avoid a feeling of social isolation during the period will be needed [4].

It should be emphasized that contact tracing and investigation for possible infection are critical to prevent the spread of infection and proper care of contacts who are infected. Therefore, after contacts are identified, it is important to investigate whether they are actually infected. This should be carried out by skilled individuals who possess interpersonal skills, cultural sensitivity, and an understanding that strict confidentiality is mandatory to protect contact identity. These are specialized skills that must be well-trained. Contacts who might have infections should be referred for proper confirmation and management [5].

Yamamura et al. [6] in this issue investigate the infection rate among close contacts of patients with coronavirus disease in Japan. They found that the infection rate showed a rising trend from 11.1% in period 1 to 19.2% and 20.0% in periods 2 and 3, respectively. The authors speculated that one important factor of the rising trend in infection rate was the change in definition used for close contacts in Japan [6]. In addition, the variants differed between the periods [7] and some variants, such as the Omicron, have been shown to have strong immune escape [8]. The different degrees of vaccination coverage between different periods must have played a significant role in shaping the magnitude of attack rates. The use of face masks, N 95 respirators, and observation of social distancing should also be quantified.

Contact tracing and early identification and isolation of patients with suspected disease are the most important measures to control the spread of infection. However, contact tracing and isolation must be integrated with other measures such as the use of appropriate personal protective equipment (PPE) and social distancing among others [9]. For example, infected individuals should be placed in a well-ventilated single-occupancy room with a closed door and dedicated bathroom. When this is difficult, confirmed COVID-19 cases may be isolated in the same locations. Home isolation of non-severe cases is also an important strategy. Environmental disinfection in health care and home settings should be implemented [10].

Although new infections have been declining and the World Health Organization has declared that COVID-19 outbreak has reduced its importance as a global pandemic, death from COVID has been higher than the death rate from influenza infection. New strains of virus have emerged periodically with no clear seasonal patterns. In addition, the protective antibodies from vaccines and natural infections are waning. Scientific and public health experts still need to decide whether and when people should get their vaccine boosters. Some of “long COVID” cases have symptoms similar to myalgic encephalomyelitis (ME) syndrome. Others may have symptoms mimicking chronic fatigue syndrome (CFS) and postural orthostatic tachycardia (POT) syndrome. These long COVID syndromes need further scientific study to help confirm the diagnosis and proper treatment. Providers must learn to recognize these syndromes to give appropriate care for patients who might have long COVID [11].

High-quality contact tracing with other non-pharmacological preventive measures needs to be upheld to deal effectively with the evolving dimension of COVID-19 despite declining the number of new cases. High-quality contact tracing will remain the cornerstone strategy in dealing with future epidemics and pandemics if and when they occur.
